# Resistance training with soy vs whey protein supplements in hyperlipidemic males

**DOI:** 10.1186/1550-2783-6-8

**Published:** 2009-03-11

**Authors:** Carol A DeNysschen, Harold W Burton, Peter J Horvath, John J Leddy, Richard W Browne

**Affiliations:** 1 Department of Nutrition and Dietetics, State University at New York, Buffalo State College, Buffalo, NY, USA; 2 Department of Exercise and Nutrition Sciences, University at Buffalo, Buffalo, NY, USA; 3 Department of Orthopaedics, University at Buffalo, Buffalo, NY, USA; 4 Department of Biotechnical and Laboratory Sciences, University at Buffalo, Buffalo, NY, USA

## Abstract

**Background:**

Most individuals at risk for developing cardiovascular disease (CVD) can reduce risk factors through diet and exercise before resorting to drug treatment. The effect of a combination of resistance training with vegetable-based (soy) versus animal-based (whey) protein supplementation on CVD risk reduction has received little study. The study's purpose was to examine the effects of 12 weeks of resistance exercise training with soy versus whey protein supplementation on strength gains, body composition and serum lipid changes in overweight, hyperlipidemic men.

**Methods:**

Twenty-eight overweight, male subjects (BMI 25–30) with serum cholesterol >200 mg/dl were randomly divided into 3 groups (placebo (n = 9), and soy (n = 9) or whey (n = 10) supplementation) and participated in supervised resistance training for 12 weeks. Supplements were provided in a double blind fashion.

**Results:**

All 3 groups had significant gains in strength, averaging 47% in all major muscle groups and significant increases in fat free mass (2.6%), with no difference among groups. Percent body fat and waist-to-hip ratio decreased significantly in all 3 groups an average of 8% and 2%, respectively, with no difference among groups. Total serum cholesterol decreased significantly, again with no difference among groups.

**Conclusion:**

Participation in a 12 week resistance exercise training program significantly increased strength and improved both body composition and serum cholesterol in overweight, hypercholesterolemic men with no added benefit from protein supplementation.

## Background

Resistance exercise is a popular training method, approved by major medical groups including the American Heart Association, the American College of Sports Medicine [1,2] to increase muscle mass and improve blood lipid profiles. It is common for males to consume commercial protein supplements and use high intensity resistance training to develop "muscle bulk" for reasons of physical appearance, competition, and/or strength gains. Sedentary individuals may also participate in resistance training to improve physical appearance, but many initiate weight lifting programs with the goal of improving overall health and fitness. It is well documented that lean muscle mass plays a significant role in determining basal metabolic rate and, thus, daily energy expenditure [3]. Creating and maintaining sites of ATP turnover and enhancing metabolic expenditure through resistance training can help prevent an age-associated decline in metabolic rate and undesirable gains in fat mass [2,4,5]. A high percentage of body fat is associated with hyperlipidemia, a known cardiovascular disease (CVD) risk factor [3]. Given that the relative risk of CVD for physically inactive individuals versus active individuals is 1.5–2.4 and that 60% of U.S. adults do not participate in regular physical activity [6], the benefit of resistance exercise in reducing CVD risk is widely recognized and is supported by all major health organizations [2,7]. Promoting the benefits and encouraging participation in this low-cost activity could help prevent CVD and other behavior-driven chronic diseases, and may provide significant cost-savings to an over-burdened health care system.

Amino acid availability is an important regulator of muscle protein metabolism during resistance training exercise [8]. Muscle net protein balance must be positive (greater muscle protein synthesis than breakdown) to experience an increase in muscle mass, which occurs only when sufficient amino acids are available in the intracellular pool. Whey and soy are both high quality sources of protein and popular supplements in the exercise community. It has been suggested soy supplementation may reduce CVD risk, a benefit that consumption of whey protein does not provide. Both proteins are easily digestible and have similar absorption kinetics [9], but some controversy exists whether soy will support skeletal muscle protein accretion in response to resistance training as effectively as whey. Phillips et al [10] reported that whey was superior to soy in stimulating amino acid uptake during a resistance training program. More recently Anthony et al [9] observed similar protein synthesis rates in exercised skeletal muscle in rats who ingested either whey or soy protein. In addition, several human studies observed no differences in either strength gains or increases in lean mass in resistance trained subjects who supplemented their diets with either soy or whey [10-13].

While supplementation with whey protein is popular with weight lifting enthusiasts, mainly to promote gains in muscle size, supplementing with soy protein is not as common. But, because of its potential to improve blood lipid profiles [14-16] soy consumption may be more appealing to a sub-set of exercisers – those at moderate or high risk for CVD. Soy's non-essential amino acid content favors post-prandial production of glucagon, which, as opposed to insulin, down-regulates lipogenic enzymes and lowers cholesterol synthesis [17]. Soy also has a number of other physiologically active compounds with cholesterol-lowering properties such as isoflavones, fiber, and phytoestrogens [14,15,18,19]. Soy protein supplementation in combination with resistance training may promote protein (or muscle) anabolism to the same extent as supplementation with whey protein [12,20,21]; so the plant-based protein's potential to lower blood lipids and reduce oxidative stress may provide a simple, cost-effective means of reducing cardiovascular disease risk.

The purpose of this study was to document changes in strength, body composition, and blood lipid profiles in sedentary, overweight, hypercholesterolemic male subjects who participated in a 12-week resistance training program and who supplemented their usual diets with either whey or soy protein versus placebo. It was hypothesized that: 1) subjects receiving either protein supplement would have equivalent gains in both strength and lean body mass and these gains would be greater than the placebo group; 2) Subjects receiving the soy supplementation would have a significant reduction in fasting blood lipid levels versus the whey and placebo groups.

## Methods

### Subjects

Thirty two healthy males from the Western New York community volunteered to participate in the study. These men (age range 21–50 years; mean 38) were generally sedentary, overweight [BMI (body mass index) 25.0–29.9], with mild to moderate hypercholesterolemia, but otherwise in overall good health. Inclusion criteria of a general sedentary lifestyle ensured that no participant recorded a BMI above 25.0 due to significant muscle mass at the beginning of the study period. Each subject was informed of the purpose and procedures of the study, and provided informed consent in accordance with the Human Subject's Review Committee of the University at Buffalo. Criteria for inclusion were: sedentary lifestyle (none or minimal routinely planned physical activity); BMI between 25.0–29.9; normal fasting blood glucose; and two or more of the following CVD risk factors: total cholesterol 200–240 mg/dl, LDL cholesterol 130–160 mg/dl, or triglycerides 150–200 mg/dl. Exclusion criteria included any prior cerebrovascular event that required hospitalization or surgery, habitual soy consumers, smokers, orthopedic or neuromuscular disorders that precluded participation in resistive exercise training and medications that affect lipid metabolism, blood pressure or cardiac function.

### Anthropometrics

Each subject's height was measured using a stadiometer (Perspective Enterprises, Kalamazoo, Michigan) and body mass was measured on a Health-O-Meter scale (Bridgeview, Illinois). Skinfolds (tricep, supraillium, abdomen and thigh) were measured with Lange skinfold calipers (Cambridge Scientific Industries, Inc., Cambridge, Maryland). All skinfolds were measured by the same investigator utilizing the same caliper for each study subject. Measures were taken in triplicate with a 2 mm reliability range. Final skinfolds were taken without viewing initial measures to minimize experimentation bias. Percent body fat was then estimated using the 4-site formula from ACSM's Resource Manual for Guidelines for Testing and Prescription [22]. Body Mass Index was calculated as body mass in kilograms divided by height in meters squared (kg/m^2^). Waist and hip circumferences were measured using a gulick measuring tape having a calibrated tension device to the nearest .25 inch. Waist measurements were taken at the minimal circumference of the abdomen and hip circumference was measured at the maximal gluteal protrusion of the buttocks. Fat free mass was calculated as body weight minus fat mass.

### Diet Analysis

During the initial screening process subjects were instructed by a registered dietitian how to maintain proper 3-day food records. Each subject completed a food record prior to beginning the exercise program and at the end of each exercise block (every 3 weeks) for a total of 5 diet records throughout the study. Records were analyzed utilizing Nutritionist Pro software (First Databank, San Bruno, CA). Based on data from diet records, the registered dietitian provided feedback to assist each subject in maintaining a protein intake equivalent between groups to approximate 1.2 g/kg body mass/day (including the supplement).

### Experimental Protocol

Subjects were initially screened by a phone interview and eligible candidates were invited to visit the laboratory, after a 12-hour fast. Potential subjects obtained additional information about the study and reviewed and signed informed consent. Subjects provided a blood sample for a blood lipid profile and blood glucose concentration The lipid profile included total cholesterol, high and low density lipoprotein cholesterol (HDL-C and LDL-C, respectively), and triglycerides using the Cholestech L· D·X^® ^(Cholestech Corporation, Hayward, CA). Height and body mass were measured to calculate BMI. If the inclusion criteria were met the participant was scheduled for a baseline blood draw in The Center for Preventive Medicine at the University at Buffalo, after a 12 hour fast (except for water) and after abstaining from caffeine, alcohol and exercise for the previous 24 hours. During this visit, body composition was measured and each subject was given diet record forms and instructed on proper completion. Subjects were also instructed how to mix (with 8 oz water or fruit juice) and to consume individual protein packets on a daily basis. Subjects were instructed that the timing of consumption of the supplement was critical. On workout days the supplement was to be taken within 60 minutes of the scheduled workout and on "off" days, at approximately the same time of day as the workout days. Subjects were instructed to limit other soy containing products in their diet as well as to maintain protein intakes as close to 1.2 g/kg body mass/day as possible (from feedback given after analysis of each of the five 3-day diet records). The resistance exercise program was reviewed and each subject underwent a medical evaluation by a physician to determine appropriateness to participate in the study.

### Experimental Groups

A double-blind protocol was used in this study whereby subjects were randomly assigned to one of three experimental groups: RES = resistance training with carbohydrate placebo; SOY = soy supplementation (25.8 g soy protein/day containing 56.2 mg isoflavones, expressed as aglycone equivalent) + resistance training; WHEY = whey supplementation (26.6 g whey protein/day) + resistance training. Coded supplements were kindly supplied by Solae LLC (St. Louis, MO) and were prepared for distribution by a trained individual not involved with any other part of the study. The formulation was developed for maximum protein delivery with minimum caloric content. The placebo contained 25 grams of complex carbohydrates (Table 1).

**Table 1:** Supplement composition (each packet 36.5 grams)^1^

**Nutrient**	**Whey**	**Soy**	**Placebo**
Kilocalories	130.0	130.0	122.4
Protein (g)	26.6	25.8	0.6
Protein (%)	73.0	70.7	1.54
Total carbohydrate (g)	5.0	5.0	30.0
Fat, acid hydrolysis (%)	2.54	1.66	N/D^2^
Isoflavones (mg/g product)			
Total isoflavones	-^3^	2.65	-^3^
Genistein-containing compounds	-^3^	1.48	-^3^
Daidzein-containing compounds	-^3^	1.03	-^3^
Glycitein-containing compounds	-^3^	0.14	-^3^
Total aglycone equivalents	-^3^	1.54	-^3^
Genistein	-^3^	0.86	-^3^
Daidzein	-^3^	0.60	-^3^
Glycitein	-^3^	0.08	-^3^
			
Ash (%)	10.1	11.4	10.3
Moisture (%)	3.6	2.7	4.2

^1^only significant levels listed

^2^not detectable

^3^contains no isoflavones

Information provided by Solae LLC, St. Louis, MO

### Blood Analysis

Blood samples were taken at baseline, prior to entering into the exercise program, and at the end of the 12 weeks of training. A total of 21 ml of blood was drawn. Seven ml were placed into a plasma tube containing an anticoagulant agent (K_3_EDTA) and the remaining 14 ml was split between 2 serum tubes with no anticoagulant. The plasma tube was immediately placed on ice, while serum tubes were left to stand at room temperature for 30 minutes to allow for clotting. All samples were centrifuged at 4°C, 1500 × g for 10 minutes, then aliquoted and stored at -80°C until analyzed.

Blood levels of cholesterol (total, LDL and HDL) and triglycerides were analyzed by enzymatic procedures (WAKO Chemicals USA, Richmond, VA). Assays for each subject were run in duplicate on the same day with the same reagent batch. External calibrators were included on every run and the concentrations in the calibration curves encompassed the range of expected sample values. Two lyophilized quality control materials were run throughout the duration of each test to estimate intra-assay reproducibility.

### Resistance Training

Subjects began resistance training under the supervision of experienced trainers soon after their first blood draw. Subjects were required to refrain from any other exercise training to minimize confounding variables. Supervised exercise sessions were identical for each subject and were held on a 3-day-a-week cycle (48–72 hours between sessions) for a total of 12 weeks that included 4 exercise blocks. Each exercise block was 21 days in duration and provided a progressive training program (Table 2). This program was based on a similar 12-week resistance training program that produced significant increases in strength and lean body mass in males [23]. Trainers instructed subjects on proper form for each exercise to minimize variation in exercise technique. For each exercise, a 4 second count was used for the concentric phase and a 2 second count for the eccentric phase. Exercises were designed to include major muscles in the upper arm, chest, back, legs, shoulder and abdomen (Table 3).

**Table 2:** Resistance training cycle/schedule

	**Reps**	**Sets**	**Rest btw Sets**	**Total Days**
**Block 1**	8–10	2–3	1 min	21
**Block 2**	8–10	3–4	1 min	21
**Block 3**	10–12	3	up to 1 min	21
**Block 4**	10–12	4	up to 1 min	21

**Table 3:** Resistance training: muscle groups & assigned exercises

	**Muscles Involved**	**Exercise**
**Day 1 workout**	chest, triceps	bench press; squats, dumbbell bench press, shoulder press, over head press
**Day 2 workout**	back, legs, and biceps	bent over rows, lunges, 1 arm rows, upright rows, back extensions
**Day 3 workout**	legs, shoulder, abdominal	flys, step-ups, shrugs, abdominal crunches, lateral raises

A one-repetition maximum (1-RM) was calculated as recommended by The American College of Sports Medicine [24] using the Brzycki regression equation, {1 RM = weight lifted during n RM/(1.0278-.0278(n)}, at the beginning of the study and each exercise block (week 1, 4, 7, 10), as a measure of strength. Subjects were required to participate in > 80% of exercise sessions over the 12 week period. Training logs for each subject were kept by assigned trainers.

### Statistical Analysis

To evaluate the effects of resistance training and protein supplementation on changes in strength and body composition a two-way repeated-measures analysis of variance design was utilized (Sigma Stat 3.0). The Tukey's test for multiple comparisons was then conducted. P < 0.05 was considered significant.

## Results

Over the course of the study, three subjects dropped out because of the inability to schedule training sessions between employment demands and outside interests. One individual ceased participation due to relocation. Twenty-eight subjects completed the study and were included in the final statistical analysis.

### Physical Characteristics

The three groups resembled each other in most baseline physical characteristics of body weight, BMI, percent body fat, fat mass, and fat free mass. The soy group had an overall higher waist-to-hip ratio versus the whey group but neither group was different from the placebo group. All groups demonstrated a significant reduction (as per cent decrease) in waist-to-hip ratio (1.1%, p < 0.05), percent body fat (8.29%, p < 0.001) and fat mass (8.1%, p < 0.001) and a significant increase in fat free mass (2.6%, p < 0.001) over the course of the study, with no difference among groups (Table 4). As expected, there was no significant change in body weight or BMI.

**Table 4:** Body composition measures.

	**PLACEBO^1^**		**WHEY^1^**		**SOY^1^**		**P-value**
	**PRE^2^**	**POST^2^**	**PRE^2^**	**POST^2^**	**PRE^2^**	**POST^2^**	**PRE vs. POST^3^**
Body Wt (kg)	89.9 ± 3.0	90.0 ± 3.0	90.0 ± 4.4	89.5 ± 4.5	92.9 ± 2.5	93.5 ± 2.5	NS^4^
BMI (kg/m^2^)	27.9 ± 0.4	27.9 ± 0.5	28.5 ± 0.7	28.4 ± 0.8	29.4 ± 0.8	29.6 ± 0.7	NS
Waist/Hip	0.90 ± 0.010^ab^	0.89 ± 0.010	0.88 ± 0.015^a^	0.86 ± 0.020	.93 ± 0.015^b^	0.92 ± 0.013	0.034
% Body Fat	22.9 ± 1.0	20.4 ± 0.9	23.0 ± 1.0	21.3 ± 1.0	24.2 ± 1.0	22.7 ± 1.0	<0.001
Fat Mass (kg)	20.6 ± 1.2	18.4 ± 1.0	21.0 ± 1.9	19.3 ± 1.7	22.5 ± 1.3	21.3 ± 1.3	<0.001
Fat Free Mass (kg)	69.2 ± 2.5	71.6 ± 2.4	68.9 ± 2.8	70.1 ± 3.1	70.3 ± 1.8	72.1 ± 1.7	<0.001

Pre- and post-study.

^1^All values are averages ± SEM; n = 9 for placebo, n = 9 for whey, n = 10 for soy.

^2 ^Pre = baseline, prior to exercise and supplementation; post = end of 12 weeks.

^3 ^Only the P value for pre versus post, with diet groups combined are presented, since diet effects were not significant and there was no interaction between diet and time (pre versus post).

^ab ^Values with a common superscript are not significantly different, at baseline (P < 0.05).

^4^NS, P > 0.0.

### Nutritional intake

Energy, macronutrient, cholesterol, dietary fiber; and alcohol intakes pre-and post-study are shown in Table 5. Total energy consumption, total carbohydrate, total fat, saturated, monounsaturated, and polyunsaturated fatty acids, total cholesterol, dietary fiber, and alcohol did not differ significantly among treatment groups over the 12 weeks of the study. Total dietary protein, grams/kg body weight protein, percent of energy from protein, and percent of energy from carbohydrates were all significantly greater post versus pre-study (p < 0.05), but percent of energy from fat was significantly lower (p < 0.05).

**Table 5:** 3-day food intake

	**PLACEBO^1^**		**WHEY^1^**		**SOY^1^**		
	**PRE^2^**	**POST^2^**	**PRE^2^**	**POST^2^**	**PRE^2^**	**POST^2^**	**PRE vs. POST P value^3^**
Total Kcal/d	1976.5 ± 111.0	2062.1 ± 125.3	2205.6 ± 270.1	2405.0 ± 135.7	2155.6 ± 297.1	2283.1 ± 291.0	NS
Total Protein (g)/d	86.1 ± 13.9	93.7 ± 18.6	97.6 ± 14.7	116.1 ± 18.2	85.3 ± 25.5	108.2 ± 22.8	0.013
Protein (g/kg BW)/d	1.0 ± 0.2	1.0 ± 0.2	1.0 ± 0.5	1.2 ± 0.3	0.92 ± 0.3	1.1 ± 0.3	0.012
Total Protein (% energy)	17.3 ± 2.4	19.3 ± 3.8	17.7 ± 4.2	19.5 ± 3.0	16.3 ± 4.4	20.7 ± 5.7	0.010
Total CHO (g)/d	228.8 ± 19.0	244.8 ± 21.8	267.4 ± 26.6	316.3 ± 19.7	230.3 ± 39.6	243.9 ± 27.0	NS
Total CHO (% energy)	45.7 ± 8.7	49.3 ± 7.3	49.5 ± 10.7	52.6 ± 7.8	41.8 ± 10.4	44.0 ± 7.1	0.031
Total Fat (g)/d	75.6 ± 20.5	66.1 ± 19.0	81.4 ± 48.3	76.0 ± 28.5	84.6 ± 38.8	77.7 ± 35.1	NS
Total Fat (% energy)	33.9 ± 7.1	30.1 ± 6.3	31.5 ± 7.8	27.5 ± 7.5	34.7 ± 7.8	30.0 ± 6.6	0.005
Saturated Fat (g)	25.4 ± 6.4	20.5 ± 5.8	26.8 ± 18.3	24.7 ± 10.2	27.9 ± 10.6	27.1 ± 12.8	NS
MUFA(g)	19.8 ± 10.6	17.4 ± 7.5	21.7 ± 11.3	19.6 ± 8.5	27.7 ± 16.5	20.0 ± 12.2	NS
PUFA (g)	10.9 ± 6.7	10.8 ± 5.2	10.7 ± 5.9	12.4 ± 8.0	12.3 ± 10.6	12.4 ± 8.7	NS
Total Cholesterol (mg)	245.7 ± 131.2	287.2 ± 118.6	295.9 ± 203.2	269.4 ± 153.9	228.3 ± 121.8	235.1 ± 75.6	NS

^1^All values are averages ± SEM; n = 9 for placebo, n = 9 for whey, n = 10 for soy.

^2^Pre = values are based on results of one 3-day intake study completed at baseline, prior to exercise and supplementation; post = end of 12 weeks.

^3 ^Only the value for pre versus post, with diet groups combined, since the diet effects were not significant and there was no interaction between diet and time (pre versus post).

^4^NS, P > 0.05; BW, body weight.

### Strength

All groups experienced a significant increase in strength (average increase = 47%, p < 0.001) (Table 6) with no significant differences among groups. All major muscle groups including chest, triceps, back, legs, shoulder, abdomen and biceps showed an increase in strength.

**Table 6:** Strength changes

	**PLACEBO^1^**		**WHEY^1^**		**SOY^1^**		
	**PRE^2^**	**POST^2^**	**PRE^2^**	**POST^2^**	**PRE^2^**	**POST^2^**	**PRE vs. POST P value^3^**
Bench Press	72.8 ± 5.9	90.3 ± 7.5	72.4 ± 8.7	89.8 ± 8.7	74.3 ± 8.1	92.5 ± 6.5	<0.001
Squats	77.5 ± 9.0	111.2 ± 13.5	75.7 ± 8.7	115.1 ± 10.0	77.1 ± 5.5	116.0 ± 6.9	<0.001
DB Bench Press	24.6 ± 2.1	34.0 ± 2.7	24.0 ± 3.2	34.9 ± 3.1	28.1 ± 3.3	36.2 ± 3.2	<0.001
Shoulder Press	15.4 ± 1.4	24.0 ± 2.1	16.9 ± 2.4	27.6 ± 4.6	17.9 ± 2.9	23.3 ± 1.9	<0.001
Triceps	16.6 ± 1.5	28.8 ± 2.3	19.3 ± 3.3	30.2 ± 3.5	19.3 ± 2.0	28.6 ± 2.9	<0.001
Bent-Over-Row	57.3 ± 7.1	77.4 ± 5.7	55.5 ± 7.0	82.0 ± 7.2	52.8 ± 4.5	73.6 ± 3.2	<0.001
Lunges	41 ± 4.0	78.5 ± 4.8	51.6 ± 8.2	85.8 ± 9.7	43.2 ± 3.9	73.7 ± 5.9	<0.001
1 Arm Row	27.6 ± 3.0	38.9 ± 3.2	24.5 ± 3.4	40.3 ± 2.8	29.2 ± 3.5	41.8 ± 2.5	<0.001
Upright Row	43 ± 3.8	55.3 ± 3.2	46.7 ± 5.5	63.8 ± 5.8	41.2 ± 2.9	54.0 ± 2.3	<0.001
Fly	19.3 ± 1.8	30.7 ± 2.5	19.1 ± 2.6	30.4 ± 2.1	18.0 ± 1.8	28.1 ± 2.1	<0.001
Shrugs	64.9 ± 9.9	96.9 ± 10.4	68.9 ± 11.2	103.9 ± 7.5	62.3 ± 6.9	100.5 ± 7.4	<0.001
Lateral Raises	12.6 ± 1.5	16.6 ± 1.7	11.4 ± 1.2	17.0 ± 1.5	13.0 ± 1.5	21.4 ± 2.9	<0.001

^1^All values (kg) are averages ± SEM; n = 9 for placebo, n = 9 for whey, n = 10 for soy.

^2^Pre = values are at baseline, prior to exercise and supplementation; post = end of 12 weeks.

^3 ^Only the P value for the combined pre vs post data is shown, since diet had no significant effect and there was no interaction between diet and time (pre vs post).

### Serum Lipids

Twelve weeks of resistance exercise resulted in a significant (average = 5.8%) decrease in fasting total cholesterol for all groups (mean reduction = 12.6 mg/dL, ± 4.5) with no differences among groups (Table 7). However, no significant changes in triglycerides, HDL-C, or TC:HDL-C were observed in any of the groups.

**Table 7:** Fasting blood measures

	**PLACEBO^1^**		**WHEY^1^**		**SOY^1^**		**P Value**
	PRE	POST	PRE	POST	PRE	POST	PRE vs. POST^2^
Total Cholesterol (mg/dL)	209.4 ± 6.0	199.0 ± 8.8	220.3 ± 13.2	204.4 ± 6.0	211.7 ± 12.6	200.5 ± 11.6	0.012
HDL-C (mg/dL)	34.0 ± 2.2	31.1 ± 2.1	32.9 ± 2.1	32.0 ± 1.6	31.1 ± 3.4	32.8 ± 2.0	NS
Triglycerides (mg/dL)	109.0 ± 17.9	126.7 ± 12.8	104.0 ± 8.3	99.6 ± 18.1	139.0 ± 21.5	127.0 ± 12.9	NS
TC:HDL-C	6.4 ± 0.4	6.7 ± 0.6	7.0 ± 0.7	6.6 ± 0.5	7.1 ± 0.4	6.1 ± 0.3	NS
LDL-C direct:HDL-C	3.9 ± 0.3	4.0 ± 0.4	4.3 ± 0.4	4.1 ± 0.4	4.1 ± 0.3	3.7 ± 0.2	NS

^1^All values are averages ± SEM; n = 9 for placebo, n = 9 for whey, n = 10 for soy.

^2 ^Only the P value for pre versus post is shown, with diet groups combined since the diet effects were not significant and there was no interaction between diet and time (pre versus post).

NS, P > 0.05

## Discussion

The principle findings of this study were that 12 weeks of resistance exercise training significantly increased muscle strength and fat free mass and significantly decreased waist-to-hip ratio, percent body fat, and total serum cholesterol in overweight, hyperlipidemic men. All groups had an equal reduction in total cholesterol, although the ratio of LDL cholesterol to HDL cholesterol tended to improve more in the soy group. These results provide further support for a structured resistance training program to improve strength and the cardiovascular risk profile of sedentary, overweight adult men desiring to improve their overall health.

Although no significant differences were observed among groups in total cholesterol and HDL-C after 12 weeks of resistance training, the soy group showed a tendency to improve both TC:HDL-C and LDL-C:HDL-C. These values were 2.5 and 2.0 times those of the whey group, respectively. These ratios are important variables in the prediction of CVD risk [25-27]. HDL-C levels are inversely related to CVD risk because HDL-C inhibits LDL oxidation (central to the initiation and progression of atherosclerosis) and reverses cholesterol transport [28,29]. Though all experimental groups demonstrated an equal reduction in total cholesterol, it may be relevant that ratios of LDL cholesterol to HDL cholesterol improved more in the soy group.

Regional distribution of fat is an important risk factor for cardiovascular disease with central (abdominal) fat deposits posing higher risk [2]; therefore our finding of a reduction in waist to hip ratio is of significant importance. The average reductions in waist and hip circumferences were 1.4 inches and 1 inch, respectively. These reductions are not likely the result of dietary changes as there were no significant changes in total calories, total fat or body weight over the course of the 12-week study. This finding supports previous studies that show resistance training decreases abdominal adiposity and reduces the waist-to-hip ratio, although total body weight changes may be small [5,8,14]. Banz et al. [1] and Ibanez et al. [14] demonstrated a significant reduction in waist-to-hip ratio and total body fat after subjects were placed on 10 and 16 weeks of resistance exercise sessions, respectively. Campbell et al. [30] also saw significant reductions in percent body fat and fat mass and a significant increase in fat free mass after 12 weeks of resistance training with subjects either on a low protein diet (0.8 g/kg/day) or on a higher protein diet (1.62 g/kg/day) diet. Our findings agree with these studies in that major changes in body weight or BMI were not observed, despite significant reductions in fat mass and adiposity. Body weight and BMI typically do not change because of concomitant increases in muscle mass and reductions in fat mass. These results, combined with others, demonstrate the limitations inherent in using changes in BMI and body weight to track the benefits of weight management programs.

Also consistent with previous studies [1,23,30], we demonstrated a significant accretion in muscle mass in a relatively short time. The ability to maintain or increase lean body mass, especially given the progressive decline in muscle mass that normally accompanies aging, is an important contributor to lowering cardiovascular disease risk [20,29]. While the use of whey supplementation to support muscle hypertrophy has been the topic of many studies, the ability of soy protein to support lean body mass gains is controversial [4,6,9,12,19]. We were most interested, though in the potential for soy to have an added benefit for groups at risk for cardiovascular disease. Several studies have shown that soy reduces serum lipid concentrations [16,18,31,32]. Coupled with our findings and those of others [9,12,19] the combination of resistance training and dietary manipulation, as part of long-term lifestyle change, may reduce risk factors for cardiovascular disease by lowering body fat stores, increasing fat free mass (an important determinant of metabolic rate), [2,3]and improving blood lipid levels.

The absence of between-group differences in strength gains between an animal-based protein supplement (whey) and vegetable-based protein supplement (soy) agrees with other studies examining the relationship between different protein sources and improved strength with resistance training. Phillips et al [10], in a study of young, healthy men completing 12 weeks of resistance training, found no significant differences in strength gains between a milk-supplemented group, a soy protein-containing group, and an energy control group. Haub et al [13] examined different protein sources in combination with 12 weeks of resistance training in older men. Their subjects displayed increased strength, with no differences between those who consumed a meat-containing diet (57% of the protein source) versus a vegetable (soy)-based diet (53% of the protein source). Strength gains were similar among all groups in our study, indicating that adequate protein rather than the protein source is important in sustaining a positive nitrogen balance for muscle accretion to occur. It should be noted that guiding subjects in all groups to consume as close to 1.2 g/kg/day of protein was to rule out confounding variables such as an excess of protein in one or more comparisons groups (i.e. the supplemented groups). While this was the intent, it can't be ruled out that this may have brought all groups to the threshold needed to gain lean body mass on a resistance training program.

The finding of a significant decrease in total serum cholesterol but no change in LDL-C, HDL-C or triglycerides and no difference among groups is surprising. The benefits of soy supplementation on improving lipid profiles are well documented [16,31-33]. Zhan et al [32] completed a meta-analysis on 23 randomized controlled trials investigating the effects of soy protein containing isoflavones on lipid profiles. The average study length in this review was 10.5 weeks. They concluded that soy protein with isoflavones significantly reduces total cholesterol, LDL cholesterol and triglycerides and the magnitude of the effect was related to the level and duration of supplement intake, to the sex of the subjects and to initial serum lipid concentrations. Anderson et al [18] also concluded that the effects of soy on lipid profiles is most pronounced in hyercholesterolemic subjects when isoflavones in the soy supplement ranged from 40 mg/day to greater than 80 mg/day. The soy supplement in our study contained 56.2 mg of isoflavones in the aglycone form. In a recent meta-analysis of 41 randomized trials with an average study length of 10 weeks, Reynolds et al [34] found that soy supplementation was associated with a significant reduction in total cholesterol, LDL cholesterol, and triglycerides (-5.26 mg/dl, -4.25 mg/dl, -6.26 mg/dl respectively) and a significant increase in HDL cholesterol (0.77 mg/dl). In a 2006 review, Torres et al [33] suggested that soy consumption reduces the clinical and biochemical abnormalities in lipid disorder-related diseases. In contrast, a study by Ma et al [35], in which subjects consumed a milk protein supplement or a soy protein supplement, found no treatment effect on lipid profiles. The length of that particular study was five weeks, which may not have been long enough to observe an effect on serum lipid levels. It was surprising that our subjects did not have a greater improvement in serum lipids with the soy supplementation after 12 weeks. A possible explanation may be individual differences in the intestinal absorption of isoflavones. Equol is a byproduct of the bio-transformation of the isoflavone diadzein by microflora in the large intestine and is a potent antioxidant [36]. Equol is not produced in the same amount in all people in response to soy consumption. It is estimated that the range of persons in the general population that are classified as "equol producers" is 14–70% [35,36], which could contribute to the variability of the effect of soy on serum lipids. The mechanisms responsible for the isoflavone-effect on lipid profiles are not currently known but may be due to their biological similarity to estrogens and estrogen-receptor-dependent genes [14,32], to enhanced bile acid secretion [32], increasing LDL receptor activity, or to enhancement of thyroxine and thyroid-stimulating hormone [14,32].

The observation that serum triglycerides showed no significant changes over the 12 weeks of the study is consistent with previous studies [37,38]. But, subjects in the soy group exhibited a trend toward reduction (lowered by 8.6% – versus a reduction in the whey group of 4.2% and an increase in the control group of 16.2%). This trend suggests that an intervention extending beyond 12 weeks may result in significant changes. Indeed, other studies have reported a beneficial effect of soy consumption alone on serum triglycerides [18,33,34].

We attempted to eliminate diet changes other than inclusion of assigned supplements. The percent of calories derived from fat decreased significantly (p < 0.05) due to the increase in energy from protein and carbohydrates in spite of no change in total energy intake. It cannot be ruled-out that the dietary fat content played a role in improved lipid profiles but its role would be minor, at best, in view of the fact that total energy and grams of fat did not change significantly. The percent of energy from protein was expected to increase in the whey and soy supplemented groups. The reasons for the increased energy from protein in the placebo group and for energy derived from carbohydrates in all groups are unknown. Community-living subjects may have naturally chosen to alter their food choices and/or lifestyle based on their enthusiasm of improved health from participation in the study.

### Study limitations

We may not have observed significant changes in body composition and lipid profiles among the different protein supplements because of a type II error and it may be that a longer (>12 weeks) training period is required to show significant changes in body composition and in lipid ratios such as TC:HDL-C and LDL-C:HDL-C. Meta-analysis by Zhan et al [32] confirmed that improvements in HDL cholesterol with soy protein supplementation were only observed in studies > 12 weeks in duration. In addition, a diet intervention (for example, limiting daily fat calories to <25%) in combination with the resistance training may have shown more dramatic results in body composition and lipid profile changes. Another limitation that may have affected the outcome of the study was the difference in initial waist:hip. After randomized enrolment it was observed the soy group had significantly higher waist:hip than the other two groups. It may be that the effect of soy was diminished because of this discrepancy. It should be noted that individuals in the placebo group did modify their diet and this included an increased percentage of energy from protein and carbohydrate sources and a decrease percent of calories from fat sources. The results of training could also be due in part to these diet changes, however; the changes in percent of energy sources as noted in the placebo group do not typically result in such dramatic increases in strength gains.

## Conclusion

Our findings add to the growing evidence that resistance training is beneficial for reducing cardiovascular risk. Our results suggest that protein supplementation is not necessary for strength or body composition changes in overweight men consuming a diet with an adequate supply of amino acids to meet the needs for stimulation of muscle protein synthesis during resistance exercise. Resistance exercise training alone increases muscle mass and improves body composition measures in sedentary, overweight men. Soy based protein supplements appear to be as effective as animal-based protein to support strength gains. Our results also suggest that soy protein supplementation during resistance training warrants further study in larger samples over longer periods of time since previous work has shown that regular soy consumption improves lipid profiles and the insulin-to-glucagon ratio and lowers oxidative stress [3,16,17,31-34].

## Competing interests

The authors declare that they have no competing interests.

## Authors' contributions

CD and HB developed the study hypothesis, research design, data collection, analysis, and manuscript preparation. PH participated in research design, data interpretation and manuscript preparation. JL participated in subject screening, interviews and manuscript preparation. RB participated in blood collection technique, analysis and interpretation of results. All authors read and approved the final manuscript.
